# Changes in the Microbiome of *Cryptosporidium*-Infected Mice Correlate to Differences in Susceptibility and Infection Levels

**DOI:** 10.3390/microorganisms8060879

**Published:** 2020-06-10

**Authors:** Raheela Charania, Brandy E. Wade, Nina N. McNair, Jan R. Mead

**Affiliations:** 1 Department of Pediatrics, Emory University, Atlanta, GA 30033, USA; raheela.charania@emory.edu (R.C.); Brandy.Wade@va.gov (B.E.W.); nmcnair@emory.edu (N.N.M.); 2 Atlanta VA Medical Center, Decatur, GA 30022, USA

**Keywords:** *Cryptosporidium*, microbiome, short-chain fatty acid, antibiotics, cloxacillin, vancomycin

## Abstract

*Cryptosporidium* spp. are opportunistic protozoan parasites that infect epithelial cells of the small intestine, causing diarrheal illness in humans. Differences in severity may be due to the immunological status of the host, malnutrition or prior exposure but may also be due to differences in the host gut flora. We examined changes in bacterial flora following antibiotic treatment to determine how cryptosporidial infections and gut integrity were affected by alterations in the microbiome. DNA was extracted from fecal and intestinal samples during peak infection. V4 region amplicons were generated and sequenced using 16sRNA on an Illumina MiSeq. Species evenness and richness were estimated using the Shannon diversity index. There was a significant decrease in anaerobes and overgrowth of Enterobacteriaceae in mice treated with cloxacillin. We also examined levels of short-chain fatty acids in fecal samples. There was a significant decrease in acetate, propionate, and butyrate in these same mice. Concurrent with the shift in bacterial infection was a significant increase in severity of cryptosporidial infection and increase in gut permeability. Treatment with other antibiotics significantly altered the microbiome but did not change the infection, suggesting that specific alterations in the host microbiome allow for more favorable growth of the parasite.

## 1. Introduction

Cryptosporidiosis can be severe and life threatening in immunocompromised individuals [1]. It is the second major cause of diarrheal illness in children globally [2] and repeated infections can lead to long-term growth deficits and cognitive impairment [3]. It is estimated that 750,000 cases in the United States of America (USA) occur annually [4]. Currently, there is no vaccine and only one drug that is Food and Drug Administration approved for immunocompetent individuals in the USA [3]. Differences in susceptibility to and severity of *Cryptosporidium parvum* infections have been observed but are not fully understood. These differences are likely multifactorial, including immunological status of the host, previous exposure, nutrition, and genetics. Despite the fact that *Cryptosporidium* is localized to the epithelium of the intestinal tract in an environment of a trillion bacteria, there have been limited studies on the effect of the gut microbiome on infection. A disrupted gut microbiome increases susceptibility to several parasitic diseases including amoebic dysentery in children [5], *Giardia* infections [6], and malaria [7].

Conversely, parasitic infections can lead to long-term dysbiosis of the gut [8]. In one human study, stools from *C. parvum*-infected volunteers showed changes in microbiota that correlated with differences in susceptibility to infection and also shifts in metabolites [9]. In mouse models of cryptosporidiosis, resident bacterial taxa are known to play a role in resistance. Germ-free mice are more susceptible to *C. parvum* infection than mice with conventional flora [10]. *C. parvum* is capable of triggering the development of inflammatory bowel disease (IBD)-like lesions in T cell receptor alpha (TCR-α) deficient mice [11]. Interestingly, mucosal lesions are much more severe in flora-bearing TCR-α deficient mice compared to TCR-α germ-free mice [11], suggesting that certain bacteria influence the pathology of disease. Bacteria are also necessary to trigger at least one innate response through the nod-like receptor [12].

Additionally, it is not understood how the microbiome affects resistance to colonization or modulates cryptosporidial infections. Microbiome changes may occur after antibiotic or drug treatment or may be influenced by diet. Our laboratory has evaluated hundreds of drugs in vivo and found that treatment with a small but significant number of potential therapeutics and antibiotics results in increased levels (intensity) of cryptosporidial infections and may also have a significant effect on fecal microbiota [13]. The present study was undertaken to determine how changes in the microbiome affect growth of the parasite and intestinal integrity, either through changes in metabolites or alteration of key host immune responses.

## 2. Materials and Methods

### 2.1. Oocyst Preparation

*C. parvum* oocysts were a kind gift from Dr. Michael Arrowood (Centers for Disease Control and Prevention) and prepared as previously described [14]. Briefly, feces from experimentally infected neonatal bovine calves were passed through stainless steel sieves and oocysts purified through a series of two discontinuous Sheather’s sucrose gradients (1.064 specific gravity over 1.103 sp. gr.) and a final microscale cesium chloride (210.6 g/L, sp. gr. 1.15) gradient. Oocysts were stored at 4 °C in 2.5% (*w/v*) aqueous potassium dichromate until used. Before use, oocysts were extensively washed with phosphate-buffered saline (PBS) to remove potassium dichromate.

### 2.2. Cryptosporidium Parvum Mouse Infection and Antibiotic Treatment

All studies were conducted in accordance with Institutional Animal Care and Use Committee (IACUC) guidelines and according to the institutionally approved animal protocol (#V016-17, approved 6/1/17). Adult IL-12 KO C57BL/6 mice were originally purchased from Jackson laboratories (Bar Harbor, ME), and subsequently bred at the Atlanta VA Medical Center animal facility. The antibiotics evaluated included cloxacillin, vancomycin-imipenem and paromomycin (a positive control that inhibits oocyst shedding by 90–95%). Cloxacillin (TCI pharmaceuticals, Falls Church, VA, USA) and paromomycin (Sigma-Aldrich, St. Louis, MO, USA) were diluted in sterile water and administered orally at 500 mg/kg and 2000 mg/kg, respectively, by daily oral gavage dosing, beginning 1 day prior to infection. Vancomycin (Geno Technology Inc., St. Louis, MO, USA), and imipenem (Biosynth International, Itasca, IL, USA) were administered in the drinking water at 0.1 mg/mL, each 1 week prior to infection. Mice (8–10 weeks old) were inoculated orally with 1 × 10^3^ oocysts and monitored for infection by collection of fecal pellets. Fecal pellets were collected prior to infection and at 7 days after infection (peak infection). Samples were either stored in potassium dichromate, as above, for parasite quantitation or flash-frozen and stored at −80 °C for DNA analysis.

Infections were assessed by quantitating the number of oocysts in the fecal pellets after concentration and purification using sucrose gradients and a flow cytometry quantitative assay [15,16]. Briefly, fecal samples were collected from individual mice on post-infection days 5 and 7 and processed through microscale sucrose gradients in 2.0 mL microcentrifuge tubes. Samples were weighed and diluent adjusted according to the weight of fecal pellets. The partially purified stool concentrate containing oocysts was incubated for 30 min at 37 °C with 5 µL of an oocyst-specific monoclonal antibody conjugated with fluorescein isothiocyanate (OW50-FITC), fixed with 4.0% paraformaldehyde, and then analyzed by means of flow cytometry. Absolute counts were calculated from the data files as oocysts per 100 µL of sample suspension.

### 2.3. DNA Extraction, Polymerase Chain Reaction (PCR), Sequencing, and Sequence Analysis

Microbiome characterization was performed by Microbiome Insights Inc. (Vancouver, BC, Canada). Specimens were placed into a MoBio PowerMag Soil DNA Isolation Bead Plate. DNA was extracted following MoBio’s instructions on a KingFisher robot. Bacterial 16S rRNA genes were polymerase chain reaction (PCR)-amplified with dual-barcoded primers targeting the V4 region, as per the protocol of Kozich et al. [17]. Amplicons were sequenced with an Illumina MiSeq using the 250-bp paired-end kit (v.2). Sequences were denoised, taxonomically classified using Greengenes (v. 13_8) as the reference database, and clustered into 97%-similarity operational taxonomic units (OTUs) with the mothur software package (https://www.mothur.org/wiki/MiSeq_SOP; accessed Nov 2017) (v. 1.39.5) [18], following the recommended procedure. The potential for contamination was addressed by co-sequencing DNA amplified from specimens and from four each of template-free controls and extraction kit reagents processed the same way as the specimens. Operational taxonomic units (OTUs) were considered putative contaminants and were removed if their mean abundance in controls reached or exceeded 25% of their mean abundance in specimens.

### 2.4. Bioinformatics and Statistical Analysis

Alpha diversity and Shannon indices were determined for each experimental condition (anatomical sites, model, and antibiotic/drug treatment). Alpha diversity was estimated with the Shannon index on raw operational taxonomic units (OTUs) abundance tables after filtering out contaminants. Significance of diversity differences was tested by analysis of variance (ANOVA). Beta diversity estimates across samples excluded OTUs occurring in fewer than 10% of the samples with a count of less than three and computed Bray–Curtis indices. Beta diversity, emphasizing differences across samples, used non-metric multidimensional (NMDS) ordination. Variations in community structure were assessed with permutational multivariate analyses of variance (PERMANOVA) with treatment group as the main fixed factor using 4999 permutations for significance testing. Analyses were conducted in the R environment.

### 2.5. Short-Chain Fatty Acids Determination

Short-chain fatty acids (SCFAs) were measured by Microbiome Insights Inc. (Vancouver, BC, Canada). For this assay, fecal samples were homogenized in an aqueous solution and analyzed in a gas chromatograph coupled with a flame ionization detector using a Thermo TG-WAXMG A GC Column, 30 m, 0.32 mm, 0.25 µm. The method used was similar to Zhao et al., 2006 [19]. To quantify SCFAs, values for each SFCA (acetic, propionic, butyric, valeric, and heptanoic acids) were determined using a calibration curve for the concentration range of 0.015–1 mg/mL.

### 2.6. Gut Permeability Assay

Intestinal permeability was determined on the day of euthanization by measuring the amount of FITC-dextran, MW 3–5 kDa (Sigma-Aldrich, St. Louis, MO, USA), in the sera 4 h after dosing. FITC-dextran is a marker of paracellular transport and mucosal barrier dysfunction [20]. Mice were euthanized and blood was collected from the hepatic portal vein using heparinized blood collection tubes. Blood was centrifuged at 6000× *g*, 1.5 min. at 4 °C and plasma was collected. Fluorescence was measured 3× for each sample in black 96-well microtiter plates using a BioTek Microplate reader with excitation at 485 nm and emission at 535 nm. Concentrations were calculated from a standard curve.

### 2.7. Statistical Analysis for Parasite Load and Fluorescein Isothiocyanate (FITC)-Dextran Permeability Assay

Statistical analyses were conducted using GraphPad Software Inc., (La Jolla, CA, USA). Differences between treatment groups and the control groups were assessed by ANOVA (mouse infection data) or by T test (FITC-dextran permeability assay). Median absolute deviation (MAD) method [21] along with mean plus or minus three standard deviation was used to calculate statistical outliers.

## 3. Results

### 3.1. Microbiota and Infection Levels Are Altered in Response to Antibiotic Treatment

**Figure 1:**
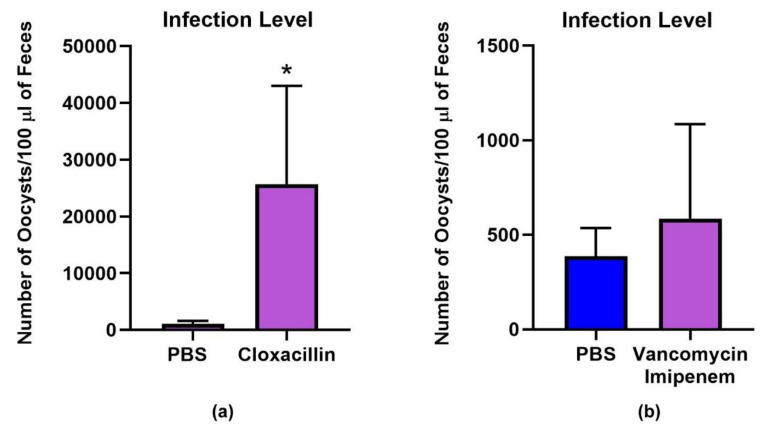
Parasite load in *C. parvum*-infected mice. Mice were infected with 10^3^ *C. parvum* oocysts and treated with either vehicle control (sterile water), cloxacillin or vancomycin-imipenem. (**a**) a significant increase in infection level was observed in the cloxacillin treated group (*p* < 0.0019, Mann–Whitney); (**b**) No significant difference was observed in mice infected with 10^3^ *C. parvum* oocysts and treated with vancomycin-imipenem.

We evaluated cloxacillin for in vivo activity, having previously demonstrated anti-cryptosporidial in vitro activity. We found that in contrast to decreasing parasite load, oral antibiotic treatment with cloxacillin significantly increased cryptosporidial infection in treated mice compared to vehicle control (Figure 1a).

**Figure 2:**
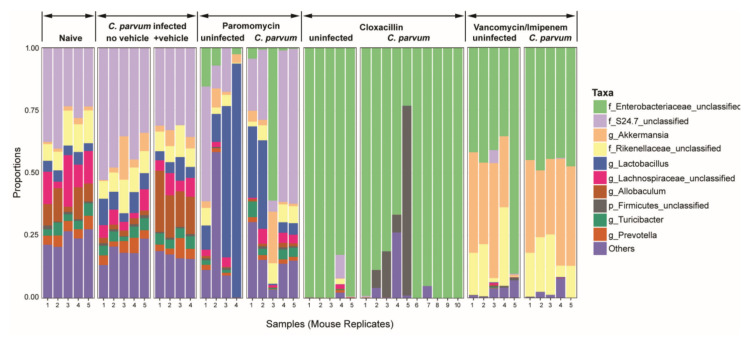
Differences in microbial composition of the microbiota in naïve and *C. parvum* infected mice including untreated and antibiotic treated groups. Relative abundances of bacterial families are shown and grouped according to their genus; bars represent individual mice within treatment groups. The antibiotics cloxacillin, vancomycin-imipenem and paromomycin disrupted the mucosal bacterial communities but only cloxacillin treatment of mice resulted in an increase in *C. parvum* infection. Statistically significant differences (Tukey′s honest significant difference (HSD)) were observed between *C. parvum* infected control and cloxacillin treatment (*p* < 0.0005), and cloxacillin treatment and naive mice (*p* < 0.0005). Paromomycin treatment alters the microbiome, but the effect on the composition was less substantial than when compared to vehicle control mice (*p* < 0.06). Decreases in *Allobaculum* and increases in *Lactobacillus*, a genus known to play a role in generating short-chain fatty acids (SCFAs), were noted. Vancomycin-imipenem treatment resulted in significant changes in the composition of the microbiome. In particular, increases in Enterobacteriaceae were observed. However, there was not a complete overgrowth of Enterobacteriaceae as observed in cloxacillin-treated mice. No statistical difference was observed between the naive (uninfected) mice and *C. parvum*-infected mice (with or without drug diluent vehicle [sterile water]).

One explanation for the increased infection level is that changes in flora were more favorable for parasite growth. Therefore, we examined differences in the microbiome that occurred in antibiotic treated mice that might be responsible for altered growth patterns. We found significant changes in relative abundance which were most altered in the cloxacillin treated mice compared to the uninfected controls (Figure 2). At the phylum level, in cloxacillin treated mice, there was almost a complete absence of *Bacteroides* (mainly consisting of anaerobes), a decrease of Firmicutes and an overgrowth of Proteobacteria. At the family level this included mainly Enterobacteriaceae (Figure 2). It should be noted that mice treated with cloxacillin displayed a distended cecum at necropsy which was slightly more severe in the infected treated group compared to the uninfected treated group.

**Figure 3:**
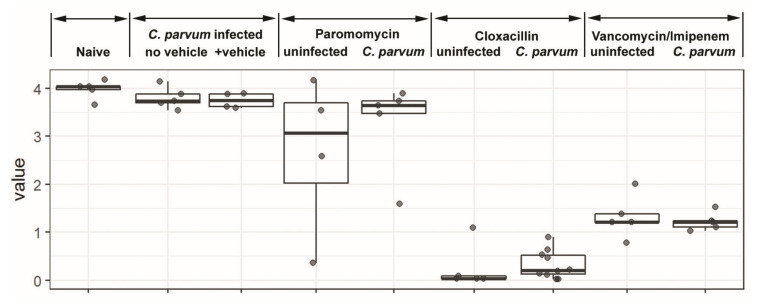
Box-plot comparisons of bacterial diversity and species richness. Bacterial diversity of the different groups using Shannon’s alpha index. The top and the bottom boundaries of each box represent the 25th and 75th quartile values. Ends of the whisker box mark the lowest and highest diversity values in each group. Cloxacillin- and vancomycin/imipenem-treated mice had significant decreases (*p* < 0.0005) in the Shannon value. The Shannon index was not significantly decreased for paromomycin treated mice vs. vehicle controls (*p* > 0.27) or naïve (*p* > 0.06) or the untreated *C. parvum* infected group alone (*p* > 0.9) when compared to uninfected or vehicle control mice (determined by analysis of variance (ANOVA)).

We examined two other antibiotics in the mouse infection model. One antibiotic, paromomycin, similar to cloxacillin, inhibits *C. parvum* in vitro [22]; [23] and additionally in vivo [23]. We found that paromomycin treatment alters the microbiome, resulting in decreases in *Allobaculum* and increases in *Lactobacillus* (Figure 2). No overgrowth of Enterobacteriaceae was observed. The overall bacterial load is decreased but the diversity is not impacted (*p* = 0.27) (Figure 3). While the microbiota is substantially altered after treatment (compared to naive, *p* > 0.06), these specific changes do not affect the gut environment in such a way as to overcome the direct inhibition the drug has on the parasite as >90% of the parasite is inhibited. Lastly, we also examined vancomycin-imipenem, an antibiotic combination that reportedly results in significant decreases in Gram positives and Gram negatives as well as a decrease in secondary fecal bile acids [24]. Vancomycin-imipenem also significantly altered the microbiome, resulting in increases in Enterobacteriaceae (but not a complete overgrowth as those observed in cloxacillin treated mice). Increases in Verrucomicrobia and decreases in Bacteroidetes were observed at the phylum level but impacted parasite growth only slightly (Figure 1b). Vancomycin-imipenem treatment resulted in a mean decrease of diversity (*p* = 0.0005) and a decrease in taxon among some samples (Figure 3).

In this study, *C. parvum* infection alone did not significantly alter flora when examined from fecal pellets (a more global assessment) (Figure 2). However, in a previous study, we found changes in flora when samples from naïve (uninfected) mice fecal pellets and infected mouse colon (*p* < 0.002) and terminal ileum were compared (*p* < 0.003, by Tukey HSD). The terminal ileum samples were more pronounced (less rich) compared to the colon which was not unexpected since the parasite infection is primarily located in this region. The most striking changes in mice infected with *C. parvum* were increased microbes belonging to the phylum Firmicutes (genera *Allobaculum* and *Turicibacter*) and decreases in the phylum Bacteroidetes (family S24.7). Thus, modest changes occur with infection in this model and were more pronounced at the local site of infection.

Species evenness was also estimated using Shannon values where a high value indicates equal distribution between species and low Shannon values suggest that certain species predominate. Significantly lower indices were found with cloxacillin treated mice when compared to vehicle control (*p* < 0.0005) (Figure 3). Decreased diversity was also observed in the vancomycin-imipenem treated mice (*p* < 0.0005), although not as severe as the cloxacillin treated mice. Shannon index was not significantly decreased for paromomycin treated mice (*p* > 0.27) or the untreated *C. parvum* infected group alone (*p* > 0.9) when compared to uninfected or vehicle control mice.

### 3.2. Gut Permeability Is Increased in Infected Mice

**Figure 4:**
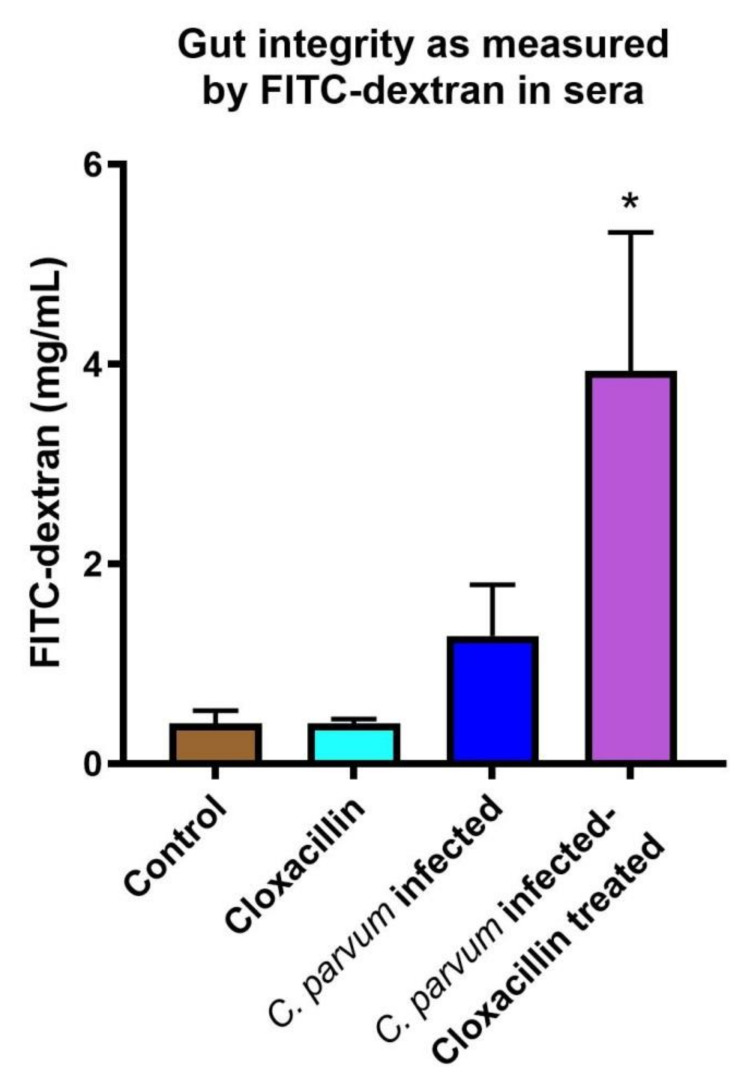
Permeability of the gut barrier. Serum FITC-dextran increased significantly in infected IL-12 KO mice treated with cloxacillin compared to uninfected controls (*p* < 0.008) as denoted by asterisk. Uninfected cloxacillin treated mice did not exhibit increased permeability.

In addition to short-chain fatty acids, we examined the role antibiotic treatment and infection levels had on gut permeability. We found that *C. parvum* infected mice had increased gut permeability. Serum levels of FITC-dextran were significantly increased (*p* < 0.008) in *C. parvum* infected mice treated with cloxacillin compared to uninfected controls (Figure 4). Cloxacillin treatment alone did not increase permeability while *C. parvum* infection resulted in only a modest increase (although not significantly due to the variability), suggesting that the higher parasite loads in the cloxacillin treated group contributed to decreased gut integrity.

### 3.3. Short-Chain Fatty Acids Are Decreased in Antibiotic Treated Mice

**Figure 5:**
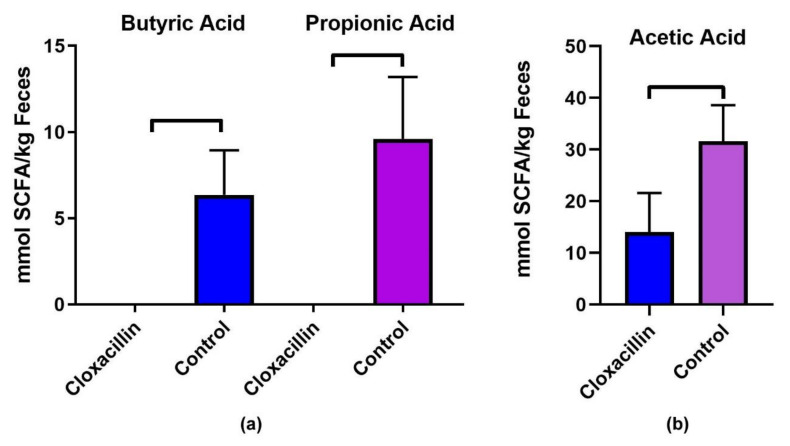
Short-chain fatty acids are decreased in antibiotic treated mice. Significant decreases in SCFCs were found in *C. parvum* infected and cloxacillin treated mice compared to *C. parvum* infected vehicle treated controls. These include (**a**) butyric (*p* = 0.005) and propionic (*p* = 0.004) acids levels below the detection limits and (**b**) levels of acetic (60% reduction compared to control, *p* = 0.009).

Since SCFAs are primarily generated by Gram-positive bacteria and can regulate T cell immune responses, we examined the changes in SCFAs in cloxacillin treated mice. Significant decreases in SCFAs were found in *C. parvum* infected and cloxacillin treated mice compared to *C. parvum* infected vehicle treated controls (Figure 5). Of the 3 major short-chain fatty acids, these included significant decreases in acetic (60% reduction compared to control, *p* = 0.009), while butyric (*p* = 0.005) and propionic (*p* = 0.004) acids were reduced to undetectable levels (Figure 5). No significant difference was observed between cloxacillin treated and control groups in levels of valeric (*p* = 0.1) and hexanoic acid (*p* = 0.18).

## 4. Discussion

Changes in the gut flora have been described in both mice [25] and monkeys [26] following *C. parvum* infection. Conversely, changes in gut flora may alter the infectivity and growth of intestinal parasites. Microbiota patterns that correlated with differences in human susceptibility to cryptosporidial infection have been described in *C. parvum* infected volunteers [9]. These differences may be inherent or influenced by diet or ingestion of certain medications. One common cause of change in the gut microbiome is through use of antibiotics. Unfortunately, antibiotic therapy may be prescribed despite not identifying the microbial agent present and may not always be appropriate for diarrhea-causing pathogens like *Cryptosporidium* [27]. In fact, in some cases, inappropriate use could lead to worsening conditions.

We examined the antibiotic cloxacillin, a beta-lactamase resistant penicillin, that was first identified in an in vitro screen for fatty acyl-CoA binding protein (CpACBP1) inhibitors [28]. It has in vitro activity against *C. parvum* with an EC_50_ of 36 µM. Despite its in vitro anti-cryptosporidial activity, we observed significantly increase infections in mice. One possible explanation was that alterations in microbiota provide favorable conditions for the parasite. In particular, host commensal microbes may limit the degree of colonization, while depletion of certain populations may allow other opportunistic bacterial pathogens or commensal flora to flourish. We observed an almost complete overgrowth of Enterobacter and a depletion of Gram-positive bacteria including Clostridiales, Erysipelotrochales and *Lactobacillus*. Similar decreases in Clostridiales and *Lactobacillus* in *T. gondii* and *Giardia muris* infected C57BL/6 mice were observed while Erysipelotrochales and *Enterobacter* was increased in acute mouse models [29].

We also examined 2 other antibiotics: 1) a vancomycin-imipenem combination with activity against a broad spectrum of both Gram-positive and Gram-negative bacteria including *Enterococcus* and *Pseudomonas* and is reported to significantly reduce overall bacterial load [30], and 2) paromomycin, a broad spectrum aminoglycoside antibiotic that inhibits protein synthesis. A previous study reported that heavy cryptosporidial infections were associated with increased abundance of Proteobacteria [31]. While increases in Enterobacteriaceae were observed in the microbiome of vancomycin-imipenem treated mice, there was not complete overgrowth as seen with cloxacillin. Populations of *Akkermansia* and Rikenellaceae still remain. Consequently, infection levels were not significantly altered. Like cloxacillin, paromomycin also has activity against *Cryptosporidium* in vitro. In paromomycin treated mice the microbiome was significantly altered, but unlike the changes observed in the cloxacillin treated mice, bacterial alterations did not seem to change the parasite infection levels as the inherent anti-cryptosporidial activity decreased parasite load by approximately 90%. While substantial decreases in the overall bacterial load were observed, no overgrowth of *Enterobacter* occurred and Gram-positive bacteria were still present. Gram-positive bacteria like *Lactobacillus* are reported to aid in maintaining the gut ecosystem and to reduce pro-inflammatory responses [32] while Clostridia species are known to play a role in generating metabolites such as butyrate, a product of fermentation [33]. Our observations would suggest that these bacteria may be important in maintaining gut homeostasis and keeping the cryptosporidial parasite in check during infection.

Little is known about metabolic changes during cryptosporidial infections and how this may impact the host immune response. *C. parvum*-infected human volunteers showed that at least one metabolite, indole, generated by indole-producing bacteria, was associated with susceptibility to *C. parvum* infection [9]. Recent attention has been focused on SCFAs that are generated primarily by Gram-positive bacteria such as Clostridia [33] by fermenting fiber. One study in mice suggested a lack of fiber may increase susceptibility to infection [31].

Concurrent with decreases in Gram-positive bacteria in cloxacillin treated mice, we observed significantly decreased levels of short-chain fatty acids. These include butyrate, propionate, and acetate, the main SCFAs produced in the gut. SCFAs have been reported to regulate the activity of T reg cells and the polarization of CD4^+^ into Th17 and Th1 effector cells that generate IFN-γ [34]. IFN-γ is an important immune factor that functions in control of cryptosporidiosis [35]. Butyrate regulates gut bacterial ecology while propionate upregulates NF-κB and IL-18 [36]. Butyrate is also a histone deacetylase inhibitor and is involved in cell cycle and cell proliferation [37]. Interestingly, butyrate, propionate and acetate are involved in upregulation of IL-18; propionate through the activation NF-κB, butyrate through the GR109 and GR43 receptors [38] and acetate through the GR43 pathway, stimulating potassium efflux and leading to inflammasome activation [38]. IL-18 is an important immune mediator in cryptosporidiosis and decreased levels of this cytokine result in increased susceptibility and infection levels [39,40]. Along with the shift in microbiota diversity, *C. parvum* infection increased significantly (Figure 2), suggesting certain commensals (e.g., Gram positives) may function in colonization resistance either directly or through the metabolites they generate.

We also wanted to examine the effect of alterations in the microbiome and infection on gut permeability since butyrate producing bacteria are known to enhance intestinal epithelial integrity [41]. We found that cloxacillin treatment by itself had little effect on gut permeability nor did vehicle control in treated mice. However, increased *Cryptosporidium* infection levels in mice, due to cloxacillin treatment, significantly increased gut permeability. Subsequent studies in our laboratory with IFN-γ KO mice (which have higher parasite loads than IL-12 KO mice) have demonstrated significantly increased FITC-dextran levels in sera (data not shown). Increased permeability was observed in *C. parvum* infected cells using transwells [42] and *C. parvum* infection of Caco-2 cells has been reported to decrease levels of the tight junction proteins, occludin and claudin 4 [43]. Interestingly, increased levels of indole increase epithelial integrity [44]. It may be that specific microbiota profiles lead to resistance to *C. parvum* infection and to more resilient gut integrity.

It is clear from the present study that specific alterations in the host microbiome allow for more favorable growth of cryptosporidial parasites. Whether this is through decreases in short-chain fatty acids or other metabolites that may be altered along with flora changes is still unclear. Future studies are needed to help identify the specific bacteria that may be responsible for increased resistance or susceptibility to infection.
